# Site-specific quantification of lysine acetylation in the N-terminal tail of histone H4 using a double-labelling, targeted UHPLC MS/MS approach

**DOI:** 10.1007/s00216-016-9431-1

**Published:** 2016-03-11

**Authors:** Annalisa D’Urzo, Alexander P. Boichenko, Thea van den Bosch, Jos Hermans, Frank Dekker, Vincenza Andrisano, Rainer Bischoff

**Affiliations:** Department of Analytical Biochemistry, University of Groningen, Antonius Deusinglaan 1, 9713 AV Groningen, The Netherlands; Department for Life Quality Studies, Alma Mater Studiorum—University of Bologna, Corso di Augusto, 237-47921 Rimini, Italy; Department of Pharmaceutical Gene Modulation, University of Groningen, Antonius Deusinglaan 1, 9713 AV Groningen, The Netherlands

**Keywords:** Histone acetylation, Tandem mass spectrometry, Multiple reaction monitoring (MRM), Post-translation modification (PTM), Histone deacetylase (HDAC) inhibitors

## Abstract

We developed a targeted liquid chromatography-tandem mass spectrometry (LC-MS/MS) method for the site-specific quantification of lysine acetylation in the N-terminal region of histone H4 by combining chemical derivatization at the protein and peptide levels with digestion using chymotrypsin and trypsin. Unmodified ε-amino groups were first modified with propionic acid anhydride and the derivatized protein digested with trypsin and chymotrypsin. The newly formed peptide N-termini were subjected to a second derivatization step with d_6_- (heavy) or d_0_- (light) acetic acid anhydride. Samples were mixed at different ratios and peptides monitored by multiple reaction monitoring (MRM) LC-MS/MS. The method was validated in terms of linearity (*R*^2^ ≥ 0.94), precision (RSD ≤ 10 %), and accuracy (≤27 %) and used to assess the effect of the histone deacetylase (HDAC) inhibitors SAHA and MS-275 in the murine macrophage-like cell line RAW 264.7. SAHA and MS-275 showed site-specific effects on the acetylation levels of K5 and K8 with the K5(Ac)–K8 and K5–K8(Ac) peptides increasing 2.5-fold and 5-fold upon treatment with SAHA and MS-275, respectively. Assessing lysine acetylation in a site-specific manner is important for gaining a better understanding of the effects of HDAC inhibitors and for clarifying disease mechanisms where lysine acetylation plays a role.

**Electronic supplementary material:**

The online version of this article (doi:10.1007/s00216-016-9431-1) contains supplementary material, which is available to authorized users.

## Introduction

In eukaryotes, the basic repeating unit of chromatin is composed of 147 bp of DNA wrapped around an octamer of two molecular groups of four histone core proteins: H2A, H2B, H3, and H4. The core histones (∼11–17 kDa) are basic proteins with a globular domain and a flexible N-terminal extension protruding from DNA, referred to as the N-terminal tails. The N-terminal regions of histones are affected by different enzyme-mediated post-translational modifications (PTMs) that change the accessibility of DNA to transcription factors and subsequent protein-protein interactions. In particular, acetylation of K5, K8, K12, and K16 in the N-terminal tail of histone H4 has been described to play an important role in the epigenetic regulation of cellular events with relevance for diseases like cancer and Alzheimer’s disease (AD). For example, histone acetylation has been shown to be crucial in hippocampal long-term potentiation (LTP) and memory formation in mice and both aging and AD pathology are associated with a loss of acetylation at the N-terminal tail of histone H4 [1, 2]. Therefore, quantification of histone acetylation on individual lysine residues is of crucial importance to understanding their role in cell biology and disease mechanisms. Next to this, quantifying histone acetylation will assist in understanding the effects of drugs such as histone deacetylase (HDAC) inhibitors, many of which are currently in clinical development.

One of the challenges in quantifying the level of acetylation at individual lysine residues in the N-terminal tail of histone H4 is the occurrence of multiple lysines within a short stretch of amino acids. While immunological approaches are useful to investigate histone modification patterns, it is technically challenging to generate antibodies that can distinguish between closely related epitopes [3]. Mass spectrometry (MS) has emerged as a powerful method to characterize histone PTMs complementary to antibody-based approaches [4]. Currently, there are two complementary approaches in proteomics: the top-down and the bottom-up approaches. In bottom-up, proteins of interest are first digested with a proteolytic enzyme and the resulting peptides analyzed by HPLC-MS/MS whereas, in top-down, the entire protein or a large fragment thereof is analyzed by HPLC-MS/MS [5]. While the top-down approach opens the possibility to gain an overview of different forms of a protein due to post-translational modifications (PTMs), it has certain limitations and presents significant challenges. Fragmentation efficiency of high molecular weight ions is poor even though fragmentations induced by electron capture dissociation (ECD) or electron transfer dissociation (ETD) are more efficient than the most widely used collision-induced dissociation (CID). Further challenges are the fact that large precursor ions are distributed across many charge states, reducing the overall sensitivity of the top-down approach. The combination of the current lack of highly efficient chromatographic separations for proteins when compared to UHPLC of peptides and the need for high-resolution, expensive mass spectrometers renders the top-down proteomics approach less suitable for the quantitative bioanalysis of protein modifications in complex mixtures. Nevertheless, it is to be expected that top-down protein analysis will gain ground with the advent of affordable high-resolution mass spectrometers, more efficient protein separation techniques, and a better understanding of the charging mechanism in electrospray ionization. With the advent of multiple reaction monitoring (MRM) for protein analysis with the bottom-up approach, it is possible to address each modification site individually on suitable signature peptides.

Here, we describe a targeted LC-MS/MS method for the site-specific quantification of lysine acetylation in the N-terminal region of histone H4 using a total histone preparation from the murine macrophage-like cell line RAW 264.7. We labelled at protein level the ε-amino groups of lysine residues with propionic acid anhydride causing a mass shift of +56 Da and protecting them from further proteolytic digestion. Although there are many examples in the literature describing methods for the site-specific quantification of lysine acetylation following the scheme of labelling with propionic acid anhydride and proteolytic digestion [6–8], unequivocal and direct site-specific quantification is difficult when multiple lysines occur in close proximity to each other in a short amino acid sequence. In order to solve this issue, we combined the labelling with propionic acid anhydride at protein level with double digestion using chymotrypsin and trypsin followed by derivatization of the neo-N-termini of suitable signature peptides with d_6_- (heavy) or d_0_- (light) acetic acid anhydride. LC-MS/MS in the multiple reaction monitoring (MRM) mode was applied to monitor changes in the acetylation level of individual lysine residues upon administration of the histone deacetylase (HDAC) inhibitors SAHA and MS-275 to RAW 264.7 cells.

## Experimental section

### Chemicals

Formic acid, hydrogen peroxide, chymotrypsin, trifluoroacetic acid (TFA), d_6_ and d_0_ acetic acid anhydride, propionic acid anhydride, ammonium hydrogencarbonate, 1.0 M triethylamine, and 16.3 M hydroxylamine were purchased from Sigma-Aldrich (St. Louis, MO, USA). Trypsin was purchased from Promega (Fitchburg, WI, USA). HPLC supra gradient acetonitrile was obtained from Biosolve (Dieuze, France). Triethylammonium hydrogencarbonate buffer, 1 M at pH 8.5, was prepared by titration of a 1-M triethylamine solution with CO_2_. Water was purified by a MilliQ Advantage A10 Water System (Millipore, Billerica, MA, USA).

### Cell culture and histone isolation

The murine macrophage-like cell line RAW 264.7 (American Type Culture Collection, Manassas, VA, USA) was cultured in Dulbecco’s modified Eagle medium (DMEM) containing 10 % heat-inactivated fetal bovine serum (FBS), 50 IU/mL penicillin, and 50 IU/mL streptomycin at 37 °C in a humidified atmosphere containing 5 % CO_2_. Cell culture reagents were purchased from Life Technologies (Carlsbad, CA, USA). For the experiments, cells were used until passage 15. One day after seeding, cells were treated with the HDAC inhibitors MS-275 (Axon Medchem, Groningen, The Netherlands) or suberoylanilide hydroxamic acid (SAHA, Selleckchem, Munich, Germany) (see Electronic Supplementary Material (ESM) Fig. S1 for structures). Inhibitor stock solutions were prepared at 10 mM for MS-275 and 4.1 mM for SAHA, respectively, in dimethylformamide (DMF), and subsequently diluted in DMEM culture medium. Cells were incubated with 1 μM of MS-275 or 0.41 μM of SAHA for 20 h, conditions that were non-toxic as determined by MTS assays (ESM Figs. S2 and S3). As a control, cells were treated with 0.01 % DMF, corresponding to the same percentage used for treatment with inhibitors. Subsequently, cells were harvested, washed with PBS, and pelleted by centrifugation at 1000 rpm for 5 min. Histones were extracted as previously described [9]. Briefly, cell pellets were suspended in 0.5 mL ice-cold buffer containing 13 mM EDTA in 10 mM Tris-base, pH 7.4. After centrifugation, sulfuric acid 0.4 M was added to the pellet with incubation of 1 h. Acetone was added to the supernatant to a final concentration of 86 % and left at −20 °C overnight to precipitate proteins. After centrifugation, acetone was removed and the pellet, dried at room temperature, was redissolved in phosphate-buffered saline (PBS, PAA Laboratories GmbH, Pasching, Austria). Total protein concentration was determined using the microBCA assay according to the manufacturer’s instructions (Pierce, Rockford, USA). Absorbance was measured with a Fluostar Optima plate reader (BMG, Labtech) at 580 nm. A bovine serum albumin standard (2 mg/mL; Pierce, Rockford, USA, # 23209) was used to calibrate the assay.

### Chemical derivatization of enriched histones

Ten micrograms of enriched and dried histones was diluted with 10 μL water and treated with 2.8 μL of 1.5 % aqueous (*w*/*v*) H_2_O_2_ for 10 min at room temperature to oxidize methionine residues to their sulfoxides [10]. Unmodified ε-amino groups were propionylated by adding 9 μL of propionic acid anhydride in 60 μL of 1 M triethylammoniumhydrogencarbonate buffer, pH 8.5. The reaction was conducted at room temperature for 10 min with mixing at 450 rpm. The obtained samples were dried under vacuum (Eppendorf vacuum concentrator, Hamburg, Germany) and dissolved in 250 mM ammoniumhydrogencarbonate, pH 8.5. Histones were digested in two different ways: with trypsin and with a combination of trypsin and chymotrypsin. Digestion with a combination of the two proteases was performed by treating histones for 6 h with 7.5 μL of chymotrypsin solution (0.2 μg/μL) at 37 °C with continuous mixing at 450 rpm and then adding 2.5 μL of trypsin solution (0.2 μg/μL) for 10 h at 37 °C. Digestion with only trypsin was performed by treating histones with 2.5 μL of trypsin solution (0.2 μg/μL) for 16 h at 37 °C (mixing at 450 rpm for all reactions). The digestions were stopped by adding 70 μL of 1 % aqueous (*v*/*v*) formic acid, and the digests were dried under vacuum. The digests were dissolved by adding 120 μL of 1 M triethylammoniumhydrogencarbonate at pH 8.5 followed by acetylation of the newly formed free N-termini with 54 μL d_0_- or d_6_- acetic acid anhydride at room temperature and mixing at 450 rpm for 10 min. The reaction was repeated three times to assure completeness. The samples were dried and then diluted in 30 μL 250 mM ammoniumhydrogencarbonate, pH 8.5. Propionylation or acetylation of hydroxyl-containing residues was reversed by hydrolyzing the ester bonds with 30 μL of 0.5 mg/mL aq. hydroxylamine for 120 min at room temperature with mixing at 450 rpm. The obtained samples were dried and dissolved in 200 μL of 1 % (*v*/*v*) aq. formic acid. Changes in histone H4 acetylation upon HDAC inhibitor treatment were assessed by mixing extracted histones from untreated cells (heavy labelled) with histones from inhibitor-treated cells (light labelled) at a 1:1 protein ratio. Method linearity was assured by analyzing control samples labelled with d_0_- or d_6_- acetic acid anhydride and mixing them at the following ratios: 0:1; 0.1:1; 0.25:1; 0.5:1; 0.75:1; 1:1; 1.5:1; 2:1, and 4:1. The final volume of all solutions was 60 μL of which 2 μL was injected for LC-MS/MS analysis in the MRM mode.

### Targeted LC-MS/MS in the MRM mode

MRM quantification was performed on a nanoAcquity UPLC system with a reversed-phase guard column (Trap Symmetry, C18, 100 Å, 5 μm, 300 μm × 50 mm) coupled to a XEVO TQ-S triple quadrupole mass spectrometer equipped with an IonKey interface (Waters, Milford, MA, USA) (HSS T3, 130 Å, 1.8 μm, 150 μm × 10 cm) using positive electrospray ionization (ESI^+^). The vaporizer temperature of the ESI^+^ source was set to 150 °C, the capillary voltage was 3.2 kV, the cone voltage was 40 V, the source offset was 50 V, the cone gas flow was 20 L/h, and the collision gas (argon) flow was set at 0.15 mL/min. Two microliters of sample solution was injected with the autosampler that was kept at 10 °C and chromatographically separated on the IonKey system at 40 °C at a flow rate of 3 μL/min. Mobile phase A was 0.1 % aq. formic acid (*v*/*v*) and mobile phase B was 0.1 % formic acid in acetonitrile. Gradient elution was started at 5 % mobile phase B and linearly increased to 100 % B at a slope of 4 % B/min. For MRM analysis, a set of two peptides comprising 8 forms corresponding to different acetylation patterns were monitored for samples digested with trypsin and chymotrypsin (ESM Table S1) and 1 peptide (16 different forms) was monitored for samples digested only with trypsin (ESM Table S2).

To establish the MRM method, transitions for all peptide forms were selected with the help of Skyline (MacCoss Lab, software version 2.6.0.6851) including the following modifications: lysine propionylation and N-terminal acetylation with d_0_- and d_6_- acetic acid anhydride. Three characteristic peptide transitions, which allowed differentiating between different modification sites, were monitored for each peptide form with the dwell time set at 10 ms. The most intense singly charged y-fragment ion was selected as quantifier. The corresponding chromatographic peak areas were integrated with Skyline and relative quantification based on the d_0_-/d_6_- ratio.

## Results and discussion

### Methodology

**Fig. 1:**
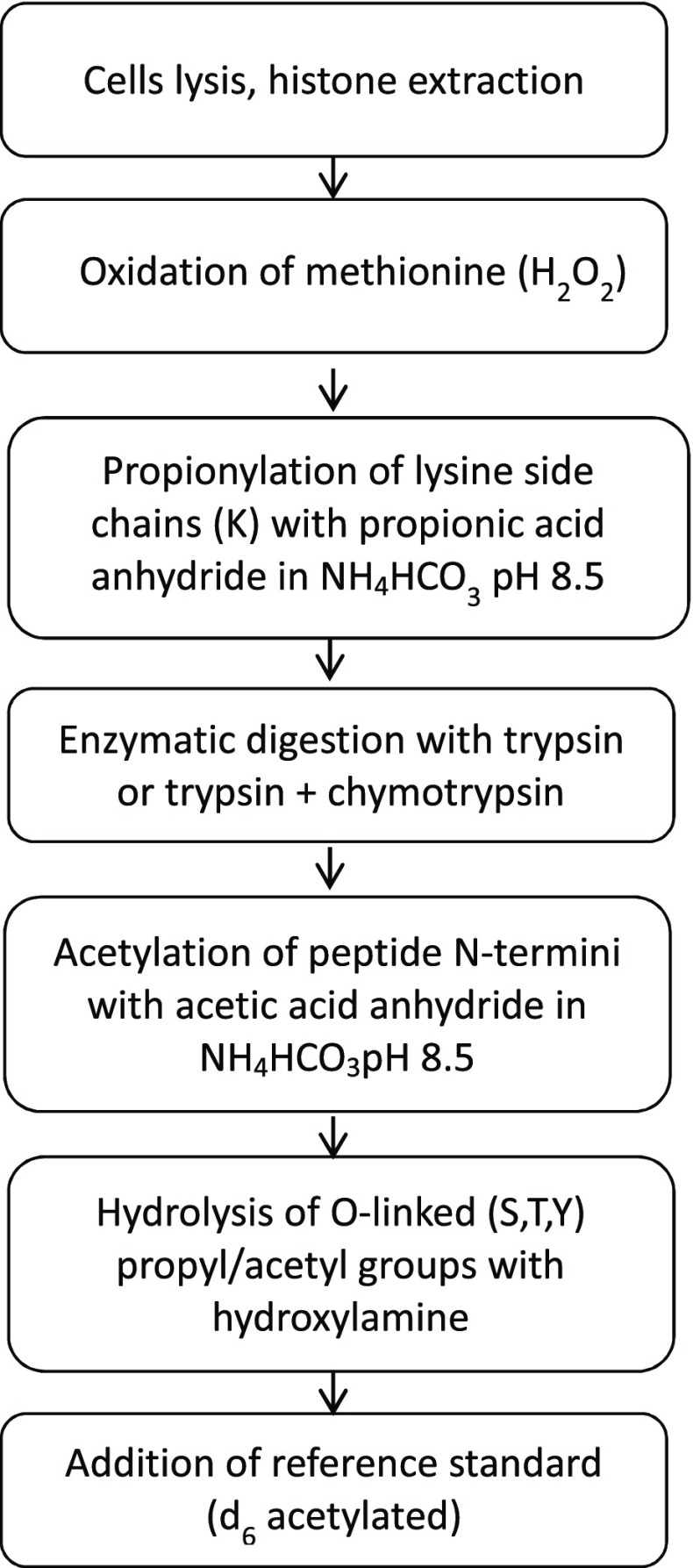
Scheme of the sample preparation procedure for the site-specific quantification of lysine acetylation in the N-terminal tail of murine histone H4 obtained from RAW 264.7 cells

A schematic overview of the methodology to monitor the lysine acetylation level in the N-terminal region of histone H4 site-specifically is given in Fig. 1. The method starts with extracting histones from cell nuclei to reduce sample complexity. While other nuclear proteins, such as ribosomal proteins, are co-extracted, the chosen signature peptides from the N-terminus of histone H4 were predicted to be unique. To render our methodology suitable for monitoring the acetylation level in methionine-containing signature peptides in future applications, we included an oxidation step with hydrogen peroxide in the procedure to fully oxidize methionine residues to their corresponding sulfoxides. Methionine-containing signature peptides are normally not preferred because they are susceptible to varying degrees of oxidation, which may affect precision and accuracy due to poor control of the ratio between the non-oxidized and mono-oxidized sulfoxide forms. To avoid such complications, we fully oxidized the methionine residue with hydrogen peroxide to its sulfoxide as previously described [10]. Propionic acid anhydride has been used as derivatization reagent to acylate amino groups at N-termini and the Ɛ-position of unmodified or mono-methylated lysines, causing a mass shift of +56 Da and protecting these residues from tryptic digestion. Moreover, since the mass difference between a propionyl (+56 Da) and the naturally occurring acetyl group on lysine residues (+42 Da) is 14 Da, it is possible to distinguish between lysines that were acetylated *in vivo* and those that were chemically derivatized. To assure that all free primary amino groups were fully propionylated, we repeated the propionylation step three times [6–8]. Because of lysine propionylation, trypsin cuts only after arginine residues resulting in a single proteolytic fragment from the amino-terminal tail of histone H4 encompassing all four lysine residues ((GKGGKGLGKGGAKR (K5–K16)), sequence (sp|P62806|H4_MOUSE histone H4 OS=*Mus musculus*). The challenge of site-specific quantification of lysine acetylation at the N-terminus of histone H4 is thus related to distinguishing between the acetylation state of K5, K8, K12, and K16. Taking all permutations into account, the total number of possible acetylated forms for this peptide is 16 (1 form for 4K-Ac, 4 forms for 3K-Ac, 6 forms for 2K-Ac, 4 forms for 1K-Ac, and 1 form without any acetylation). However, only some of these forms can be unambiguously quantified due to overlapping MRM transitions (ESM Table S2). In order to resolve this problem, we combined propionylation of lysine residues at the protein level with proteolytic digestion using a combination of trypsin and chymotrypsin to cleave the peptide (GKGGKGLGKGGAKR) also after leucine. Each of the formed peptides, GKGGKGL and GKGGAKR, contains two lysine residues leading to four different acetylated forms that can be quantified unambiguously because of characteristic MRM transitions (ESM Table S1). In order to render the method suitable for relative quantification, we performed a second derivatization step after proteolytic digestion by treating the samples with either d_0_- or d_6_- acetic acid anhydride to incorporate d_0_- or d_3_-acetate at the newly formed free N-terminal amino groups, resulting in mass additions of +42 or +45 Da, respectively.

Since acid anhydrides react also with the hydroxyl groups of threonine, serine, and tyrosine, we treated the derivatized samples with hydroxylamine for selective hydrolysis of ester bonds and restoration of the free hydroxyl groups, making the method suitable to monitor peptides bearing these residues [11, 12]. Mixing the d_0_- and d_6_-labelled samples allowed relative quantification of each form of the signature peptides GKGGKGL and GKGGAKR from the N-terminus of murine histone H4.

**Fig. 2:**
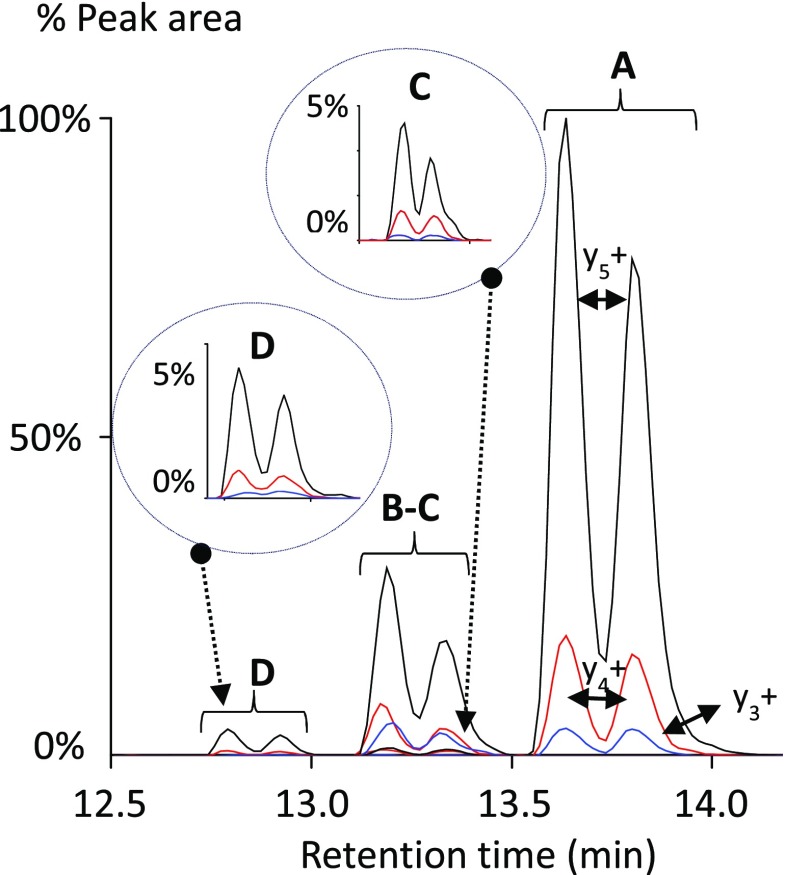
LC-MS/MS (MRM) analysis of all four possible forms of GKGGAKR due to different acetylation patterns (**A**–**D**). Three transitions were monitored for each peptide form, two qualifiers (y_4_+, y_3_+), and one quantifier (y_5_+). **A** MRM d_0_-GKGGAKR; **B** MRM d_0_-GK(+42)GGAK(+56)R; **C** MRM d_0_-GK(+56)GGAK(+42)R; **D** MRM d_0_-GK(+42)GGAK(+42)R. *Circle C* enlargement of the MRM trace of d_0_-GK(+56)GGAK(+42)R, which is present at lower intensity and overlaps with the MRM trace of d_0_-GK(+42)GGAK(+56)R (**B**). *Circle D* enlargement of the MRM trace of d_0_-GK(+42)GGAK(+42)R (**D**). *Black traces* y_5_+; *red traces* y_4_+; *blue traces* y_3_+ (see Table S-1 for details)

Figure 2 shows the LC-MS/MS chromatograms of GKGGAKR (quantifier transition (y_5_+) and qualifier transitions (y_4_+, y_3_+) monitoring each of the four d_0_-forms related to the pattern of acetylation at K12 and K16. The four forms eluted within a time window of less than two minutes and peptides with a higher degree of propionylation eluted at increased retention times as expected [13]. Surprisingly, almost all MRM LC-MS/MS chromatograms for different transitions showed double peaks as shown in Fig. S4 (see ESM). Since double peaks were also found for peptides without lysine residues, we hypothesized that they originated from chemical derivatization of the N-terminus with acetic acid anhydride after proteolytic digestion due to racemization of the C-terminal amino acid via oxazolone formation [14, 15] (for more details, see ESM Figs. S5 and S6 and the accompanying text). The method may be extended to monitor lysine acetylation in other regions of histone H4, other histones, or other proteins that may be acetylated such as the transcription factor NF-ƙB. It must, however, be considered that MRM on quadrupole mass analyzers has limited mass resolution compared to high-resolution mass analyzers (e.g., TOF or Orbitrap). This implies, for example, that lysine trimethylation (+42.04 Da) cannot be distinguished from lysine acetylation (+42.01 Da). Although trimethylation is not a known modification at the N-terminus of histone H4, it may interfere at other sites. In this case, synthetic peptides carrying the anticipated modification will have to be used to ascertain that the proper signature peptide is being monitored, since the retention times of peptides containing acetylated versus trimethylated lysine residues will differ.

### Method validation

The method was validated with respect to precision and accuracy by mixing the (d_0_-/d_6_-)labelled histone H4-derived signature peptides at ratios ranging from 0:1 to 4:1. Regression lines were linear across the measured range with correlation coefficients of 0.94–0.98 (ESM Table S3), and the retention times were similar for both d_0_- and d_6_-labelled peptides (see ESM Fig. S4). Intra-day and inter-day precision for histone H4-derived peptides after combined trypsin and chymotrypsin digestion was determined at two (d_0_-/d_6_-) ratios, analyzing six replicates within the same day or spread over three different days. The relative standard deviation for the inter-day precision was below 0.26 % for the retention time (<0.16 s) and below 10.1 % with respect to peak area (Tables 1 and 2). Accuracy of the method was estimated to be better than 27 % by comparing peak areas of peptides labelled with d_0_- and d_6_- acetic acid anhydride and mixed at a 1:1 ratio (ESM Table S4).

**Table 1:** Precision of peak areas for histone H4-derived peptides after chymotrypsin and trypsin digestion analyzing six replicates spread over three different days

Measured peptide forms	Average peak area (*n* = 18)	Relative standard deviation (%)
[+d_0_-/d_6_-]GK[+56.0]GGK[+56.0]GL	0.452	0.18
[+d_0_-/d_6_-]GK[+56.0]GGAK[+56.0]R	0.441	0.55
[+d_0_-/d_6_-]GK[+42.0]GGK[+56.0]GL	0.502	3.15
[+d_0_-/d_6_-]GK[+56.0]GGK[+42.0]GL	0.493	10.09
[+d_0_-/d_6_-]GK[+42.0]GGAK[+42.0]R	0.540	0.13
[+d_0_-/d_6_-]GK[+42.0]GGAK[+56.0]R	0.498	3.44
[+d_0_-/d_6_-]GK[+56.0]GGAK[+42.0]R	0.489	0.64
[+d_0_-/d_6_-]GK[+56.0]GGK[+56.0]GL	0.215	2.46
[+d_0_-/d_6_-]GK[+56.0]GGAK[+56.0]R	0.212	0.69
[+d_0_-/d_6_-]GK[+42.0]GGK[+56.0]GL	0.270	6.24
[+d_0_-/d_6_-]GK[+56.0]GGK[+42.0]GL	0.268	6.39
[+d_0_-/d_6_-]GK[+42.0]GGAK[+42.0]R	0.264	1.98
[+d_0_-/d_6_-]GK[+42.0]GGAK[+56.0]R	0.241	3.82
[+d_0_-/d_6_-]GK[+56.0]GGAK[+42.0]R	0.236	0.60

The levels refer to the following (d_0_-/d_6_-) mixing ratios: 0.5:1 (upper part) and 0.3:1 (lower part)

**Table 2:** Precision of retention times for histone H4-derived peptides after chymotrypsin and trypsin digestion analyzing six replicates spread over three different days

Measured peptide forms	Average retention time (*n* = 18)	Relative standard deviation (%)
[+d_0_-/d_6_-]GK[+56.0]GGK[+56.0]GL	17.688	0.028
[+d_0_-/d_6_-]GK[+56.0]GGAK[+56.0]R	13.610	0.000
[+d_0_-/d_6_-]GK[+42.0]GGK[+56.0]GL	16.848	0.010
[+d_0_-/d_6_-]GK[+56.0]GGK[+42.0]GL	17.077	0.044
[+d_0_-/d_6_-]GK[+42.0]GGAK[+42.0]R	12.781	0.096
[+d_0_-/d_6_-]GK[+42.0]GGAK[+56.0]R	13.176	0.039
[+d_0_-/d_6_-]GK[+56.0]GGAK[+42.0]R	13.180	0.000
[+d_0_-/d_6_-]GK[+56.0]GGK[+56.0]GL	17.694	0.045
[+d_0_-/d_6_-]GK[+56.0]GGAK[+56.0]R	13.611	0.014
[+d_0_-/d_6_-]GK[+42.0]GGK[+56.0]GL	16.853	0.034
[+d_0_-/d_6_-]GK[+56.0]GGK[+42.0]GL	17.091	0.265
[+d_0_-/d_6_-]GK[+42.0]GGAK[+42.0]R	12.780	0.117
[+d_0_-/d_6_-]GK[+42.0]GGAK[+56.0]R	13.167	0.101
[+d_0_-/d_6_-]GK[+56.0]GGAK[+42.0]R	13.181	0.015

The levels refer to the following (d_0_-/d_6_-) mixing ratios: 0.5:1 (upper part) and 0.3:1 (lower part)

### Evaluation of HDAC inhibitors

**Fig. 3:**
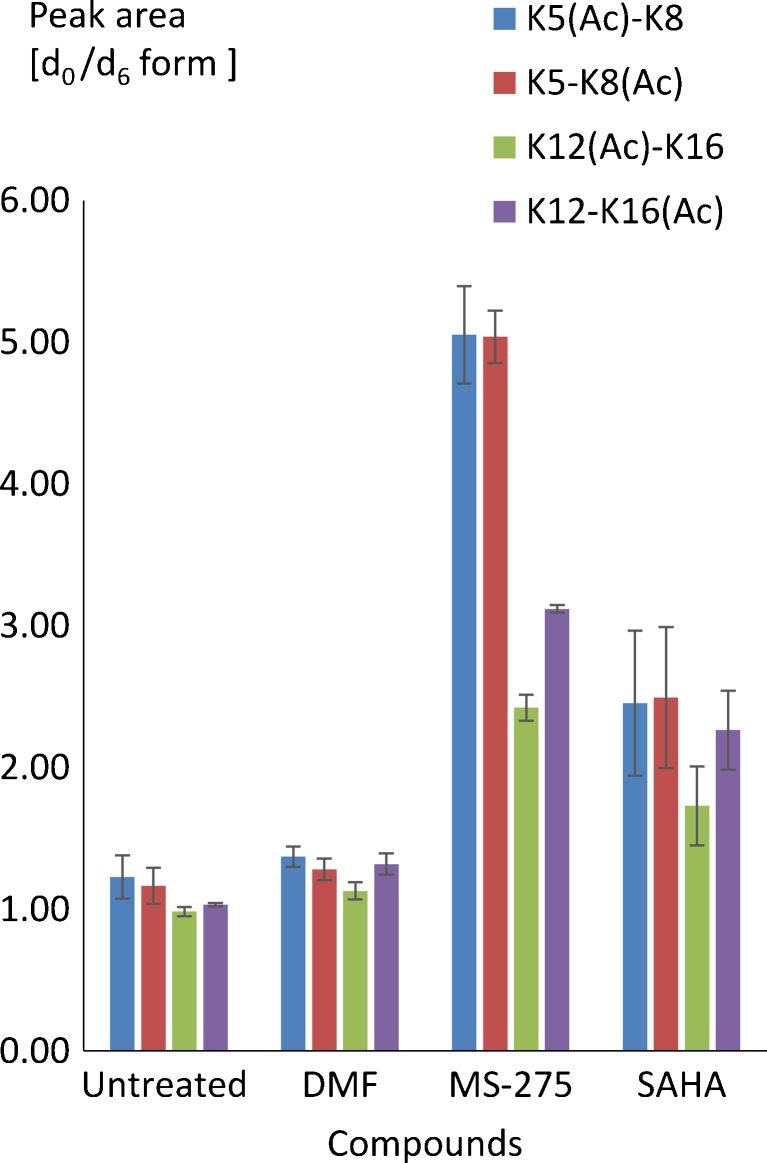
Effect of the HDAC inhibitors MS-275 (1 μM) and SAHA (0.41 μM) on lysine acetylation in the N-terminal tail of murine histone H4 upon administration to RAW264.7 cells. 0.01 % DMF was included as control to mimic the effect of the solvent on histone acetylation. Acetylated lysine residues are indicated (*Ac*). The standard deviation relates to three independent biological replicates each analyzed twice. Statistically significant differences (*p* < 0.05) were found when comparing each monitored form of MS-275- and SAHA-treated sample with the corresponding forms from the DMF-treated sample (MS-275-DMF and SAHA-DMF) and comparing each form between the two inhibitor-treated cells (MS-275-SAHA); see ESM Table S6 for more details on how the peak areas were calculated with the corresponding statistical parameters

MS-275 and SAHA are two structurally distinct orally active HDAC inhibitors that are in clinical use (SAHA for cutaneous T cell lymphoma) or are currently being studied in clinical trials for the treatment of certain types of cancer [16], inflammation [17], viral infections [18], and neurodegeneration [19]. We applied the developed methodology to determine the site-specific effect of MS-275 and SAHA on the acetylation status of K5, K8, K12, and K16 in the N-terminal region of histone H4 upon administration to RAW 264.7 murine macrophages. Macrophages play a key role in inflammatory responses, and while the treatment of inflammatory diseases is a potential area of application of HDAC inhibitors, the effect of HDAC inhibitors on the site-specific acetylation of histones in macrophages has not been reported. SAHA was administrated at 0.41 μM (limited by cellular toxicity) and MS-275 at 1 μM, both concentrations that are above the IC_50_ values of these inhibitors for class I HDACs except for HDAC8 in the case of MS-275 (ESM Table S5). A histone extract from untreated cells (d_6_-labelled) was mixed 1:1 with an extract from treated cells (d_0_-labelled) and the d_6_- to d_0_- peak area ratios for the peptides GKGGKGL (K5–K8) and GKGGAKR (K12–K16) monitored the different peptide forms to assess changes in lysine acetylation levels. Treatment of RAW264.7 cells with MS-275 and SAHA resulted in increased acetylation at all lysine residues (Fig. 3). Treatment with MS-275 led to a 5-fold increase in acetylation at K5(Ac)–K8 and K5–K8(Ac), respectively, while this increase was about 2.5-fold for SAHA. Acetylation of K12(Ac)–K16 and K12–K16(Ac) was increased by approximately 2–2.5-fold for both inhibitors (*p* < 0.05). The fully acetylated forms were not detected.

The higher level of K5(Ac)–K8 and K5–K8(Ac) for MS-275-treated cells is in agreement with previous findings, albeit in different cells, indicating that treatment with this inhibitor leads to more robust hyper-acetylation than that with SAHA [20, 21].

## Conclusions

The analytical strategy of chemical derivatization at the protein and peptide levels, combined with digestion using chymotrypsin and trypsin, allows differentiating between the acetylation levels at individual lysine residues. We demonstrate the utility of this method by analyzing changes in the lysine acetylation profile of the N-terminal region of histone H4 upon treatment of RAW 264.7 cells with the HDAC inhibitors MS-275 and SAHA showing that MS-275 results in significantly higher levels of acetylation at K5(Ac)–K8 and K5–K8(Ac) than SAHA. The described methodology may be adapted to monitor site-specific lysine acetylation changes in other histones as well as in non-histone proteins.

## Electronic supplementary material

Below is the link to the electronic supplementary material.

ESM 1 (PDF 671 kb)
