# Supplementary material for: Site-specific quantification of lysine acetylation in the N-terminal tail of histone H4 using a double-labelling, targeted UHPLC MS/MS approach
Source: Anal Bioanal Chem. 2016 Mar 11;408:3547–53. doi: 10.1007/s00216-016-9431-1 (PMC4837199; doi:10.1007/s00216-016-9431-1)
Supplement: Supplementary file 1 — (PDF 671 kb) [file 216_2016_9431_MOESM1_ESM.pdf]

Analytical and Bioanalytical Chemistry

Electronic Supplementary Material

Site-specific quantification of lysine acetylation in the N-terminal tail of histone H4 using a double-labelling, targeted UHPLC MS/MS approach

Annalisa D'Urzo, Alexander P. Boichenko, Thea van den Bosch, Jos Hermans, Frank Dekker, Vincenza Andrisano, Rainer Bischoff

Table of Contents

Table S1	p. S-3
Table S2	p. S-4, S-5, S-6
Table S3	p. S-7
Table S4	p. S-8
Table S5	p. S-9
Table S6	p. S-9
Figure S1	p. S-10
Figure S2	p. S-11
Figure S3	p. S-12
Figure S4	p. S-13
Figure S5	p. S-14
Figure S6	p. S-15
References	p. S-16

Table S1 Overview of all possible acetylated forms of the peptides GKGGKGL and GKGGAKR following derivatization with propionic acid anhydride, combined chymotrypsin and trypsin digestion and acetylation of the N-terminal region of histone H4 with d-0 acedic acid anhydride. The MRM transitions allow the unequivocal quantification of all forms. +42.0 stands for an acetylation, +56.0 for propionylation. and d-$_0$ for acetylation N-terminus with acedic acid anhydride (light)

Peptide	Forms	Precursor (m/z) 1^+ charge state	Product (m/z)	
GKGGKGL	d_0-GK[+56.0]GGK[+56.0]GL	770.44	671.41	y6
	d_0-G]K[+56.0]GGK[+56.0]GL	770.44	487.29	y5
	d_0-GK[+56.0]GGK[+56.0]GL	770.44	430.27	y4
	d_0-G]K[+42.0]GGK[+56.0]GL	756.43	657.40	y6
	d_0-G]K[+42.0]GGK[+56.0]GL	756.43	487.29	y5
	d_0-GK[+42.0]GGK[+56.0]GL	756.43	430.27	y4
	d_0-GK[+42.0]GGK[+42.0]GL	742.41	643.38	y6
	d_0-GK[+42.0]GGK[+42.0]GL	742.41	473.27	y5
	d_0-GK[+42.0]GGK[+42.0]GL	742.41	416.25	y4
	d_0-GK[+56.0]GGK[+42.0]GL	756.43	657.40	y6
	d_0-GK[+56.0]GGK[+42.0]GL	756.43	473.27	y5
	d_0-GK[+56.0]GGK[+42.0]GL	756.43	416.25	y4
		**2^+ charge state**		
GKGGKGR	d_0-GK[+56.0]GGAK[+56.0]R	414.24	544.32	y5
	d_0-GK[+56.0]GGAK[+56.0]R	414.24	487.30	y4
	d_0-GK[+56.0]GGAK[+56.0]R	414.24	430.28	y3
	d_0-GK[+42.0]GGAK[+56.0]R	407.23	544.32	y5
	d_0-GK[+42.0]GGAK[+56.0]R	407.23	487.30	y4
	d_0-GK[+42.0]GGAK[+56.0]R	407.23	430.28	y3
	d_0-GK[+42.0]GGAK[+42.0]R	400.22	530.30	y5
	d_0-GK[+42.0]GGAK[+42.0]R	400.22	473.28	y4
	d_0-GK[+42.0]GGAK[+42.0]R	400.22	416.26	y3
	d_0-GK[+56.0]GGAK[+42.0]R	407.23	530.30	y5
	d_0-GK[+56.0]GGAK[+42.0]R	407.23	473.28	y4
	d_0-GK[+56.0]GGAK[+42.0]R	407.23	416.26	y3

Table S2 Overview of all possible acetylated forms of the peptide GKGGKGLGKGGAKR. Trypsin digestion of the N-terminus of histone H4 results in the signature peptide GKGGKGLGKGGAKR, which may exist in 16 different acetylated forms. Four forms with one acetylated lysine residue, six forms with two acetylated lysine residues, four forms with three acetylated lysine residues, one form with four acetylated lysine residues and one form with none acetylated lysine Green highlighting indicates MRM transitions that allow assigning acetylated forms unequivocally, while all other transitions are not unambiguous. +42.0 stands for an acetylation, +56.0 for propionylation and d-0 for acetylation N-terminus with acetic acid anhydride (light)

Peptide	Forms with zero acetylated lysine	Precursor (m/z)	Product (m/z)
1	d_0-GK[+56.0]GGK[+56.0]GLGK[+56.0]GGAK[+56.0]R	768.95	955.57
	d_0-GK[+56.0]GGK[+56.0]GLGK[+56.0]GGAK[+56.0]R	768.95	898.55
	d_0-GK[+56.0]GGK[+56.0]GLGK[+56.0]GGAK[+56.0]R	768.95	785.46
	d_0-GK[+56.0]GGK[+56.0]GLGK[+56.0]GGAK[+56.0]R	768.95	544.32
	d_0-GK[+56.0]GGK[+56.0]GLGK[+56.0]GGAK[+56.0]R	768.95	487.30
	**Forms with one acetylated lysine**		
2	d_0-GK[+42.0]GGK[+56.0]GLGK[+56.0]GGAK[+56.0]R	761.94	955.57
	d_0-GK[+42.0]GGK[+56.0]GLGK[+56.0]GGAK[+56.0]R	761.94	898.55
	d_0-GK[+42.0]GGK[+56.0]GLGK[+56.0]GGAK[+56.0]R	761.94	785.46
	d_0-GK[+42.0]GGK[+56.0]GLGK[+56.0]GGAK[+56.0]R	761.94	544.32
	d_0-GK[+42.0]GGK[+56.0]GLGK[+56.0]GGAK[+56.0]R	761.94	487.30
3	d_0-GK[+56.0]GGK[+42.0]GLGK[+56.0]GGAK[+56.0]R	761.94	955.57
	d_0-GK[+56.0]GGK[+42.0]GLGK[+56.0]GGAK[+56.0]R	761.94	898.55
	d_0-GK[+56.0]GGK[+42.0]GLGK[+56.0]GGAK[+56.0]R	761.94	785.46
	d_0-GK[+56.0]GGK[+42.0]GLGK[+56.0]GGAK[+56.0]R	761.94	544.32
	d_0-GK[+56.0]GGK[+42.0]GLGK[+56.0]GGAK[+56.0]R	761.94	487.30
4	d_0-GK[+56.0]GGK[+56.0]GLGK[+42.0]GGAK[+56.0]R	761.94	941.55
	d_0-GK[+56.0]GGK[+56.0]GLGK[+42.0]GGAK[+56.0]R	761.94	884.53
	d_0-GK[+56.0]GGK[+56.0]GLGK[+42.0]GGAK[+56.0]R	761.94	771.45
	d_0-GK[+56.0]GGK[+56.0]GLGK[+42.0]GGAK[+56.0]R	761.94	544.32
	d_0-GK[+56.0]GGK[+56.0]GLGK[+42.0]GGAK[+56.0]R	761.94	487.30
5	d_0-GK[+56.0]GGK[+56.0]GLGK[+56.0]GGAK[+42.0]R	761.94	941.55
	d_0-GK[+56.0]GGK[+56.0]GLGK[+56.0]GGAK[+42.0]R	761.94	884.53
	d_0-GK[+56.0]GGK[+56.0]GLGK[+56.0]GGAK[+42.0]R	761.94	771.45
	d_0-GK[+56.0]GGK[+56.0]GLGK[+56.0]GGAK[+42.0]R	761.94	530.30
	d_0-GK[+56.0]GGK[+56.0]GLGK[+56.0]GGAK[+42.0]R	761.94	473.28

	**Forms with two acetylated lysines**		
	d_0-GK[+42.0]GGK[+42.0]GLGK[+56.0]GGAK[+56.0]R	754.93	955.57
	d_0-GK[+42.0]GGK[+42.0]GLGK[+56.0]GGAK[+56.0]R	754.93	898.55
**6**	d0-GK[+42.0]GGK[+42.0]GLGK[+56.0]GGAK[+56.0]R	754.93	785.46
	d_0-GK[+42.0]GGK[+42.0]GLGK[+56.0]GGAK[+56.0]R	754.93	544.32
	d_0-GK[+42.0]GGK[+42.0]GLGK[+56.0]GGAK[+56.0]R	754.93	487.30
	d_0-GK[+56.0]GGK[+42.0]GLGK[+42.0]GGAK[+56.0]R	754.93	941.55
	d_0-GK[+56.0]GGK[+42.0]GLGK[+42.0]GGAK[+56.0]R	754.93	884.53
**7**	d_0-GK[+56.0]GGK[+42.0]GLGK[+42.0]GGAK[+56.0]R	754.93	771.45
	d_0-GK[+56.0]GGK[+42.0]GLGK[+42.0]GGAK[+56.0]R	754.93	544.32
	d_0-GK[+56.0]GGK[+42.0]GLGK[+42.0]GGAK[+56.0]R	754.93	487.30
	d_0-GK[+56.0]GGK[+56.0]GLGK[+42.0]GGAK[+42.0]R	754.93	927.54
**8**	d_0-GK[+56.0]GGK[+56.0]GLGK[+42.0]GGAK[+42.0]R	754.93	870.52
	d_0-GK[+56.0]GGK[+56.0]GLGK[+42.0]GGAK[+42.0]R	754.93	757.43
	d_0-GK[+56.0]GGK[+56.0]GLGK[+42.0]GGAK[+42.0]R	754.93	530.30
	d_0-GK[+56.0]GGK[+56.0]GLGK[+42.0]GGAK[+42.0]R	754.93	473.28
	d_0-GK[+42.0]GGK[+56.0]GLGK[+42.0]GGAK[+56.0]R	754.93	941.55
	d_0-GK[+42.0]GGK[+56.0]GLGK[+42.0]GGAK[+56.0]R	754.93	884.53
**9**	d_0-GK[+42.0]GGK[+56.0]GLGK[+42.0]GGAK[+56.0]R	754.93	771.45
	d_0-GK[+42.0]GGK[+56.0]GLGK[+42.0]GGAK[+56.0]R	754.93	544.32
	d_0-GK[+42.0]GGK[+56.0]GLGK[+42.0]GGAK[+56.0]R	754.93	487.30
	d_0-GK[+56.0]GGK[+42.0]GLGK[+56.0]GGAK[+42.0]R	754.93	941.55
	d_0-GK[+56.0]GGK[+42.0]GLGK[+56.0]GGAK[+42.0]R	754.93	884.53
**10**	d_0-GK[+56.0]GGK[+42.0]GLGK[+56.0]GGAK[+42.0]R	754.93	771.45
	d_0-GK[+56.0]GGK[+42.0]GLGK[+56.0]GGAK[+42.0]R	754.93	530.30
	d_0-GK[+56.0]GGK[+42.0]GLGK[+56.0]GGAK[+42.0]R	754.93	473.28
	d_0-GK[+42.0]GGK[+56.0]GLGK[+56.0]GGAK[+42.0]R	754.93	941.55
	d_0-GK[+42.0]GGK[+56.0]GLGK[+56.0]GGAK[+42.0]R	754.93	884.53
**11**	d_0-GK[+42.0]GGK[+56.0]GLGK[+56.0]GGAK[+42.0]R	754.93	771.45
	d_0-GK[+42.0]GGK[+56.0]GLGK[+56.0]GGAK[+42.0]R	754.93	530.30
	d_0-GK[+42.0]GGK[+56.0]GLGK[+56.0]GGAK[+42.0]R	754.93	473.28
	**Forms with three acetylated lysines**		
	d_0-GK[+56.0]GGK[+42.0]GLGK[+42.0]GGAK[+42.0]R	747.92	927.54
**12**	d_0-GK[+56.0]GGK[+42.0]GLGK[+42.0]GGAK[+42.0]R	747.92	870.52
	d_0-GK[+56.0]GGK[+42.0]GLGK[+42.0]GGAK[+42.0]R	747.92	757.43
	d_0-GK[+56.0]GGK[+42.0]GLGK[+42.0]GGAK[+42.0]R	747.92	530.30

	d_0-GK[+56.0]GGK[+42.0]GLGK[+42.0]GGAK[+42.0]R	747.92	473.28
**13**	d_0-GK[+42.0]GGK[+42.0]GLGK[+42.0]GGAK[+56.0]R	747.92	941.55
	d_0-GK[+42.0]GGK[+42.0]GLGK[+42.0]GGAK[+56.0]R	747.92	884.53
	d_0-GK[+42.0]GGK[+42.0]GLGK[+42.0]GGAK[+56.0]R	747.92	771.45
	d_0-GK[+42.0]GGK[+42.0]GLGK[+42.0]GGAK[+56.0]R	747.92	544.32
	d_0-GK[+42.0]GGK[+42.0]GLGK[+42.0]GGAK[+56.0]R	747.92	487.30
**14**	d_0-GK[+42.0]GGK[+56.0]GLGK[+42.0]GGAK[+42.0]R	747.92	927.54
	d_0-GK[+42.0]GGK[+56.0]GLGK[+42.0]GGAK[+42.0]R	747.92	870.52
	d_0-GK[+42.0]GGK[+56.0]GLGK[+42.0]GGAK[+42.0]R	747.92	757.43
	d_0-GK[+42.0]GGK[+56.0]GLGK[+42.0]GGAK[+42.0]R	747.92	530.30
	d_0-GK[+42.0]GGK[+56.0]GLGK[+42.0]GGAK[+42.0]R	747.92	473.28
**15**	d_0-GK[+42.0]GGK[+42.0]GLGK[+56.0]GGAK[+42.0]R	747.92	941.55
	d_0-GK[+42.0]GGK[+42.0]GLGK[+56.0]GGAK[+42.0]R	747.92	884.53
	d_0-GK[+42.0]GGK[+42.0]GLGK[+56.0]GGAK[+42.0]R	747.92	771.45
	d_0-GK[+42.0]GGK[+42.0]GLGK[+56.0]GGAK[+42.0]R	747.92	530.30
	d_0-GK[+42.0]GGK[+42.0]GLGK[+56.0]GGAK[+42.0]R	747.92	473.28
	**Forms with four acetylated lysines**		
**16**	d_0-GK[+42.0]GGK[+42.0]GLGK[+42.0]GGAK[+42.0]R	740.92	927.54
	d_0-GK[+42.0]GGK[+42.0]GLGK[+42.0]GGAK[+42.0]R	740.92	870.52
	d_0-GK[+42.0]GGK[+42.0]GLGK[+42.0]GGAK[+42.0]R	740.92	757.43
	d_0-GK[+42.0]GGK[+42.0]GLGK[+42.0]GGAK[+42.0]R	740.92	530.30
	d_0-GK[+42.0]GGK[+42.0]GLGK[+42.0]GGAK[+42.0]R	740.92	473.28

Table S3 Peptides from the N-terminus of murine histone H4 that are formed after combined proteolytic digestion with trypsin and chymotrypsin. For each peptide the four forms, corresponding to different modification patterns, are given with their corresponding MS/MS transitions, collision energies and retention times. Correlation coefficients (R2) relate to 9-point calibration curves that were generated by measuring samples that were labelled with d0- or d6-acetic acid anhydride at different mixing ratios over the range from 0:1 to 4:1. [+d0-/ d6-] stands for acetic acid anhydride (light) related to acetic acid anhydride (heavy)

Measured acetylated forms	Precursor m/z (d_0- / d_6-)	Quantifier (m/z)	Collision energy (V)	Retention time, (min)	R^2
**GKGGKGL**					
[+d_0-/ d_6-]GK[+56.0]GGK[+56.0]GL	770.44 / 773.46	487.28 (y_5)	27	17.7	0.98
[+d_0-/ d_6-]GK[+42.0]GGK[+56.0]GL	756.42 / 759.44	657.39 (y_6)	27	17.1	0.94
[+d_0-/ d_6-]GK[+56.0]GGK[+42.0]GL	770.44 / 773.46	473.27 (y_5)	27	17.1	0.97
[+d_0-/ d_6-]GK[+42.0]GGK[+42.0]GL	not detected				
**GKGGAKR**					
[+d_0-/ d_6-]GK[+56.0]GGAK[+56.0]R	414.24/ 415.75	544.32/ (y_5)	14	13.7	0.98
[+d_0-/ d_6-]GK[+42.0]GGAK[+56.0]R	407.23/408.74	544.32 (y_5)	14	13.2	0,98
[+d_0-/ d_6-]GK[+56.0]GGAK[+42.0]R	407.23/408.75	530.30 (y_5)	14	13.2	0,98
[+d_0-/ d_6-]GK[+42.0]GGAK[+42.0]R	400.22 /401.73	530.30 (y_5)	14	12.7	0.95

Table S4 Accuracy of the method was estimated by comparing the peak areas of peptides labelled with d_6- or d_0-acetic anhydride and mixed at a 1:1 ratio (combined chymotrypsin/trypsin digestion)

Peptide forms after chymotrypsin and trypsin digestion	Average area d_0-/ d_6- 1:1	Accuracy (%)
**GKGGKGL**		
[+d_0-/ d_6]GK[+56.0]GGK[+56.0]GL	1.05	5
[+d_0-/ d_6]GK[+42.0]GGK[+56.0]GL	1.19	19
[+d_0-/ d_6]GK[+56.0]GGK[+42.0]GL	0.96	4
**GKGGAKR**		
[+d_0-/ d_6]GK[+56.0]GGAK[+56.0]R	1.09	9
[+d_0-/ d_6]GK[+42.0]GGAK[+56.0]R	1.13	13
[+d_0-/ d_6]GK[+56.0]GGAK[+42.0]R	1.13	13
[+d_0-/ d_6]GK[+42.0]GGAK[+42.0]R	1.27	27

Table S5 Reported IC_{50} values for the inhibition of class I HDACs by SAHA and MS-275

HDAC inhibitor	HDACs IC_{50} (nM)				Refs
	HDAC-1	HDAC-2	HDAC-3	HDAC-8	
SAHA	21-148	144-418	6-509	38-1700	[1-4]
MS-275	181-300	306-1155	499-8000	2700->10000	[1-3,5]

µ

Table S6 Acetylation levels of peptides GKGGKGL (K5-K8) and GKGGAKR (K12-K16) from the N-terminal region of murine histone H4 upon treatment of RAW 264.7 cells with 0.41 µM SAHA or 1.0 µM MS-275 in comparison to untreated and DMF-treated controls. Results are expressed as area ratios between the d_0- (treated) versus the d_6- (untreated) cells relative to the non-acetylated form of GKGGKGL (K5-K8). The standard deviation relates to three independent biological replicates each analyzed twice. Acetylation levels between DMF-treated and inhibitor-treated cells (DMF versus MS-275) (DMF versus SAHA) and between MS-275 and SAHA-treated cells (MS -275 versus SAHA) were all significantly different ($p < 0.05$)

	K5(Ac)K8	K5-K8(Ac)	K12(Ac)-K16	K12-K16(Ac)
	d_0-/d_6[K5(Ac)K8]/ d_0-/d_6 [K5-K8]	d_0-/d_6 [K5-K8(Ac)]/ d_0-/d_6 [K5-K8]	d_0-/d_6 [K12(Ac)-K16]/ d_0-/d_6 [K5-K8]	d_0-/d_6 [K12-K16(Ac)]/ d_0-/d_6 [K5-K8]
	Area average ± SD			
Untreated	1.23 ± 0.15	1.17 ± 0.13	1.13 ± 0.03	1.03 ± 0.01
DMF	1.37 ± 0.07	1.28 ± 0.08	2.42 ± 0.06	1.32 ± 0.08
MS-275	5.05 ± 0.34	5.04 ± 0.18	2.42 ± 0.09	3.12 ± 0.03
SAHA	2.45 ± 0.51	2.49 ± 0.50	1.73 ± 0.28	2.26 ± 0.28

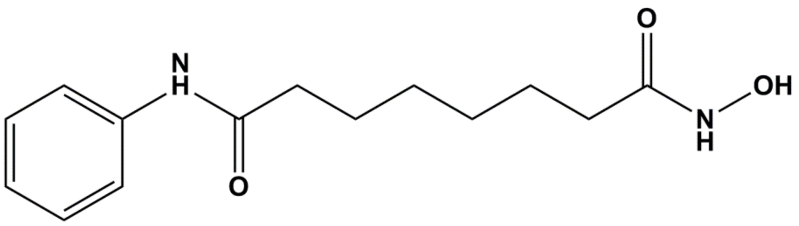

SAHA

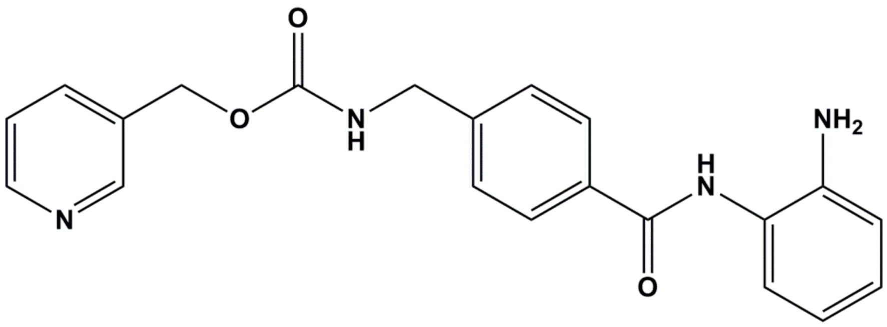

MS -275

Fig. S1 Chemical structures of SAHA and MS-275

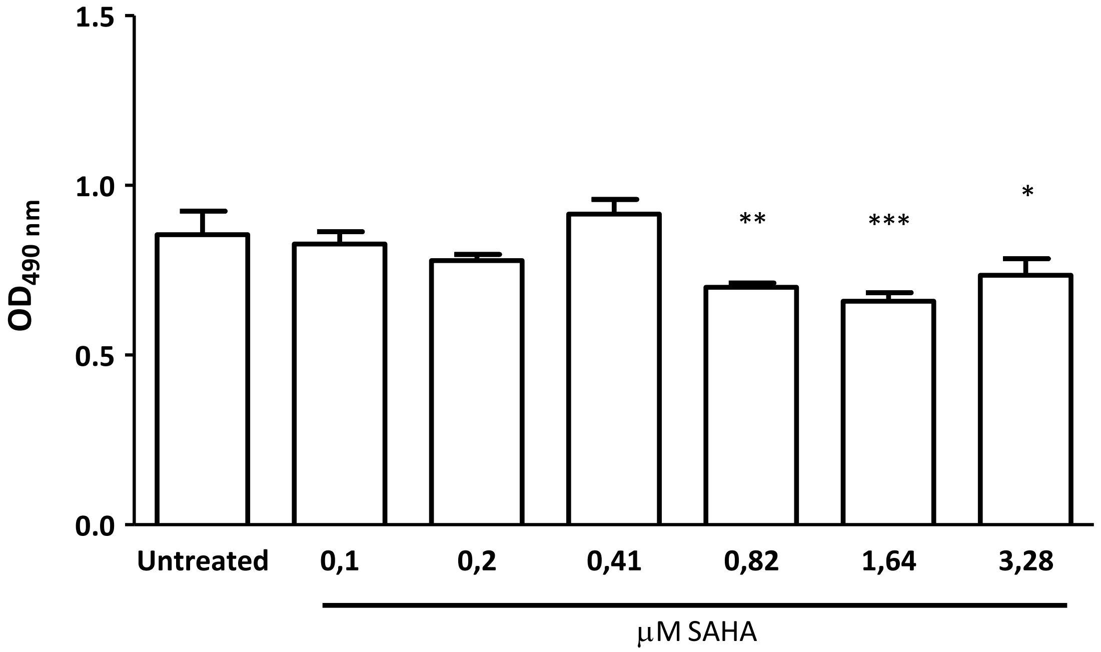

Fig. S2 Results of cytotoxicity (MTS) assays for SAHA on RAW264.7 cells. Cells were seeded at 7500 cells per well in 96-well plates. On the following day, medium was replaced with medium containing SAHA at the given concentrations. After 20 hrs incubation, the MTS reagent (Promega, Wisconsin, USA) was added to the wells. After 1 hr of incubation with MTS, the absorbance at 490 nm was measured using a plate reader. The amount of absorbance at this wavelength is indicative of mitochondrial conversion of MTS, which in turn indicates cell viability. The experiment was done in triplicate. No statistical differences were observed between untreated cells, or cells treated with SAHA up to a concentration of 0.41 μM while viability decreased significantly at higher concentrations. Hence, we conducted our studies with SAHA at 0.41 μM. *** $p < 0.001$, ** $p < 0.01$ and * $p < 0.05$ compared to untreated cells

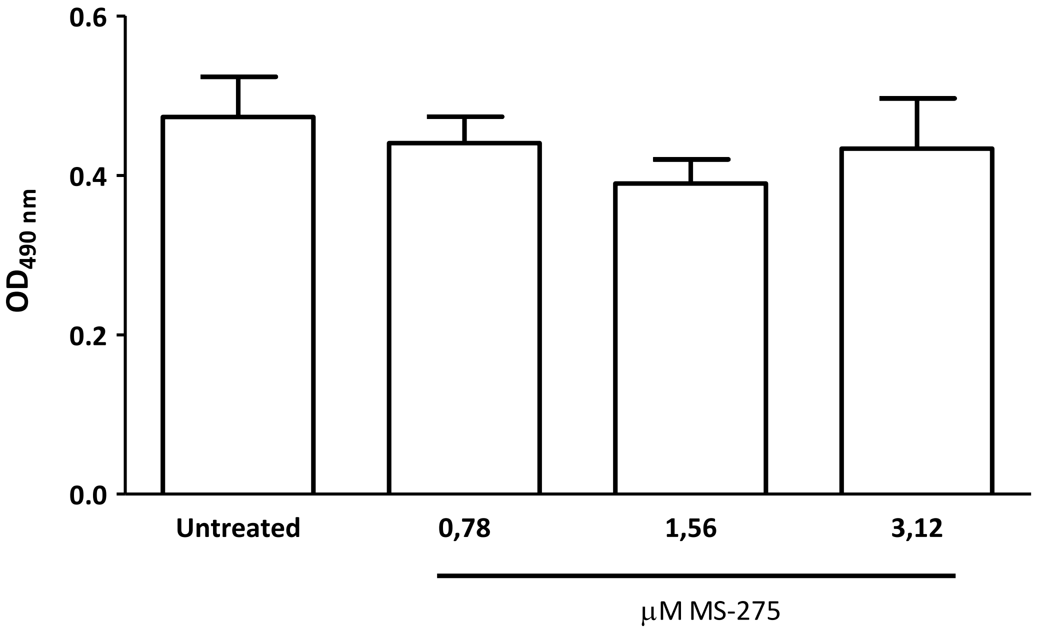

Fig. S3 Results of cytotoxicity (MTS) assays for MS-275 on RAW264.7 cells. Cells were seeded at 7500 cells per well in 96 wells plates. On the following day, medium was replaced with medium containing MS-275 at the given concentrations. After 20 hrs of incubation, the MTS reagent (Promega, Wisconsin, USA) was added to the wells. After 1 hr of incubation with MTS, the absorbance at 490 nm was measured using a plate reader. The amount of absorbance at this wavelength is indicative of mitochondrial conversion of the MTS reagent, which in turn indicates cell viability. The experiment was done in triplicate. No statistical differences were observed between untreated cells, or cells treated with MS-275 up to a concentration of 3.12 μM. Hence, we concluded that there is no cytotoxicity of MS-275 to RAW264.7 cells at these concentrations

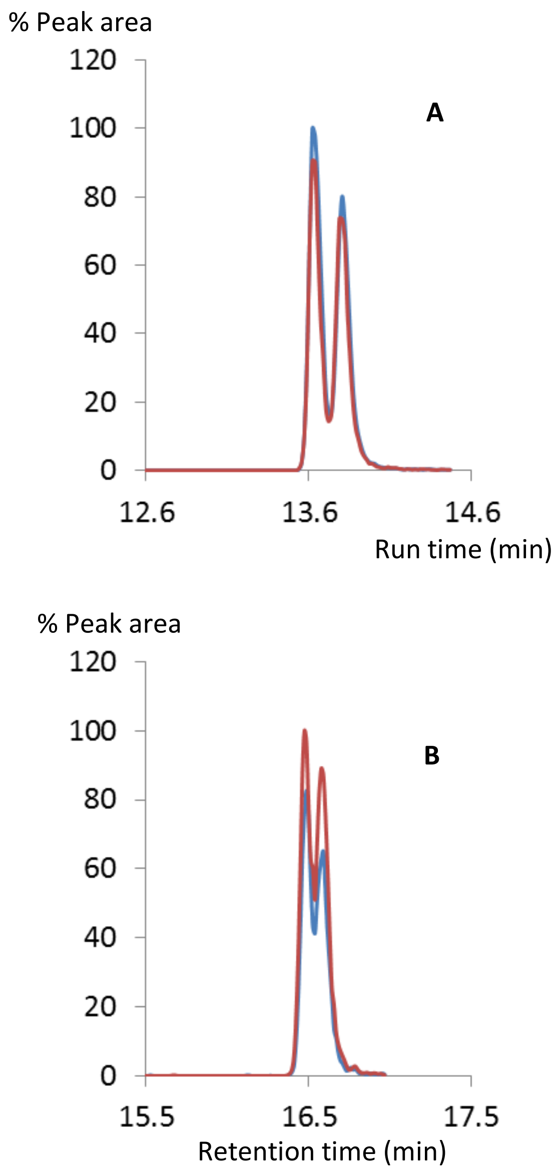

Fig. S4 LC-MS/MS (MRM) analysis of signature peptides from the N-terminus of histone H4. Panel A: Peptide GKGGAKR (SRM transitions: d_0-407.23 m/z^{2+} → d_0-544.329 $m/z\ y_5^{1+}$ and d_6-408.74 m/z^{2+} → d_6-544.329 $m/z\ y_5^{1+}$) after digestion of histone H4 with chymotrypsin and trypsin. Panel B: peptide GKGGKGLGKGGAKR (SRM transitions: d_0-768.94 m/z^{2+} → d_0-544.32 m/z^{1+} and d_6-770.45 m/z^{2+} → d_6-544.32 m/z^{1+}) after digestion of histone H4 with trypsin. Blue traces: d_0- N-terminally acetylated forms, red traces: d_6- N-terminally acetylated forms

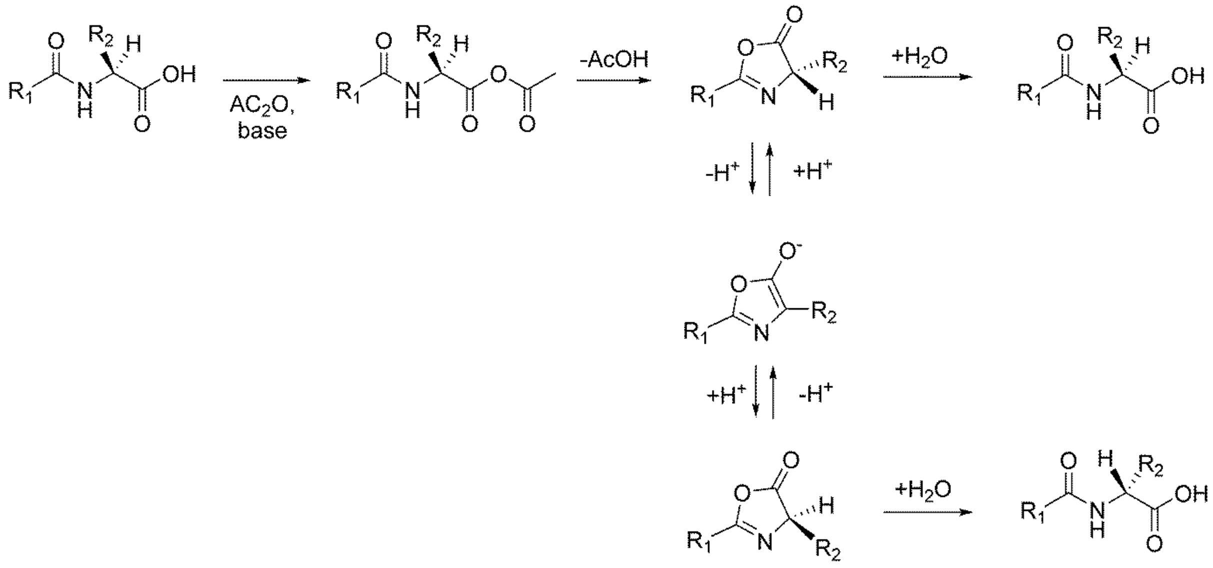

Figure S5 Mechanism of formation of two diastereoisomeric peptides via an oxazolone intermediate. Acetic acid anhydride may activate the C-terminal carboxylic acid group in the form of a mixed anhydride, which can rearrange to an oxazolone intermediate that is prone to racemization. Subsequent hydrolysis of the oxazolone gives two diastereoisomeric peptides which can be chromatographically separated resulting in double peaks (see Fig. S4). The proposed reaction mechanism suggests a major role for the acetylation conditions and the nature of the base that is used during the acetylation reaction, with more nucleophilic bases facilitating the rearrangement[6,7]. To find support for this hypothesis, we monitored the peptide GKGGKGLGKGGAKR after trypsin digestion of histone H4 by MRM LC-MS/MS. Ammonium-ion-containing buffers such as triethylammonium hydrogen carbonate (1 M, pH 8.5) resulted in double peaks and repeated additions of acetic acid anhydride led to an increase in the intensity of the second peak (Fig. S6, panels B and C). This phenomenon was less pronounced upon acetylation in sodium carbonate buffer (1 M, pH 8.5) (Fig. S6, panel A). Both findings are in line with the hypothesis that oxazolone formation triggers racemization of the C-terminal amino acid as described by Anderson et al. and Bodanszky at al., and that the nucleophilicity of the base plays an important role in the racemisation process. We therefore concluded that the observed double peaks are related to diastereoisomeric forms of the same peptide and integrated the areas from both peaks for the quantitative analysis of site-specific histone H4 acetylations

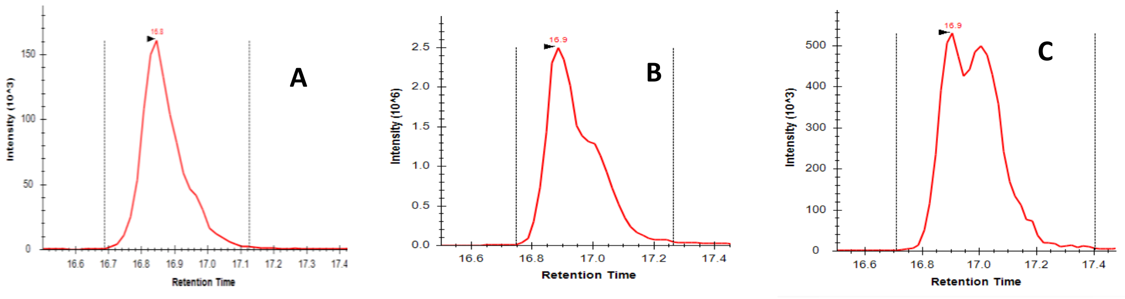

Fig. S6 Intramolecular rearrangement of N-terminally acetylated peptides via oxazolones under different digestion conditions. LC-MS/MS (MRM) analysis of the histone H4-derived peptide GKGGKGLGKGGAKR after trypsin digestion and acetylation step. The acetylation reaction with d_0-acetic anhydride was performed in two different buffers each at 1 M concentration and pH 8.5 over increasing reaction times at room temperature.

Panel A: Sodium carbonate (3 consecutive treatments with acetic acid anhydride for 10 min).

Panel B: Triethylammoniumhydrogencarbonate (3 consecutive treatments with acetic acid anhydride for 10 min).

Panel C: Triethylammoniumhydrogencarbonate (4 consecutive treatments with acetic acid anhydride for 20 min)

References

1. Khan N, Jeffers M, Kumar S, Hackett C, Boldog F, Khramtsov N, Qian X, Mills E, Berghs SC, Carey N, Finn PW, Collins LS, Tumber A, Ritchie JW, Jensen PB, Lichenstein HS, Sehested M (2008) Determination of the class and isoform selectivity of small-molecule histone deacetylase inhibitors. Biochemical Journal 409:581-589
2. Beckers T, Burkhardt C, Wieland H, Gimmnich P, Ciossek T, Maier T, Sanders K (2007) Distinct pharmacological properties of second generation HDAC inhibitors with the benzamide or hydroxamate head group. International Journal of Cancer 121 (5):1138-1148
3. Ning Z-Q, Li Z-B, Newman MJ, Shan S, Wang X-H, Pan D-S, Zhang J, Dong M, Du X, Lu X-P (2012) Chidamide (CS055/HBI-8000): a new histone deacetylase inhibitor of the benzamide class with antitumor activity and the ability to enhance immune cell-mediated tumor cell cytotoxicity. Cancer Chemotherapy and Pharmacology 69 (4):901-909
4. Wei DG, Chiang V, Fyne E, Balakrishnan M, Barnes T, Graupe M, Hesselgesser J, Irrinki A, Murry JP, Stepan G, Stray KM, Tsai A, Yu H, Spindler J, Kearney M, Spina CA, McMahon D, Lalezari J, Sloan D, Mellors J, Geleziunas R, Cihlar T (2014) Histone Deacetylase Inhibitor Romidepsin Induces HIV Expression in CD4 T Cells from Patients on Suppressive Antiretroviral Therapy at Concentrations Achieved by Clinical Dosing. Plos Pathogens 10 (4)
5. Hu ED, Dul E, Sung CM, Chen ZX, Kirkpatrick R, Zhang GF, Johanson K, Liu RG, Lago A, Hofmann G, Macarron R, de los Frailes M, Perez P, Krawiec J, Winkler J, Jaye M (2003) Identification of novel isoform-selective inhibitors within class I histone deacetylases. Journal of Pharmacology and Experimental Therapeutics 307 (2):720-728
6. Anderson GW, Zimmerma.Je, Callahan FM (1967) A Reinvestigation of Mixed Carbonic Anhydride Method of Peptide Synthesis. Journal of the American Chemical Society 89 (19):5012-&
7. Bodanszky M, Ondetti MA (1966) Peptide Synthesis. Interscience Publishers, New York
